# Human limbal fibroblast-like stem cells induce immune-tolerance in autoreactive T lymphocytes from female patients with Hashimoto’s thyroiditis

**DOI:** 10.1186/s13287-017-0611-5

**Published:** 2017-07-03

**Authors:** Antonina Coppola, Laura Tomasello, Maria Pitrone, Salvatore Cillino, Pierina Richiusa, Giuseppe Pizzolanti, Carla Giordano

**Affiliations:** 1 Laboratory of Regenerative Medicine, Section of Endocrinology, Diabetology and Metabolism, Di.Bi.M.I.S., University of Palermo, Piazza delle Cliniche 2, 90127 Palermo, Italy; 2 ATeN (Advanced Technologies Network Center), University of Palermo, Palermo, Italy; 3 Department of Ophthalmology, University of Palermo, Palermo, Italy

**Keywords:** Human limbal stem cells, Hashimoto’s thyroiditis, Immunoregulation, Tolerance induction, Inflammatory diseases

## Abstract

**Background:**

Due to their “natural immune privilege” and immunoregulatory properties human fibroblast-like limbal stem cells (f-LSCs) have acquired great interest as a potential tool for achieving immunotolerance. Hashimoto’s thyroiditis (HT) is the most common thyroid autoimmune disease and cause of hypothyroidism. To date, conventional hormone replacement therapy and unspecific immunosuppressive regimens cannot provide a definitive cure for HT subjects. We explored the immunosuppressant potential of human f-LSCs on circulating lymphomonocytes (PBMCs) collected from healthy donors and female HT patients.

**Methods:**

We assessed the immunophenotyping of f-LSCs, both untreated and after 48 h of proinflammatory cytokine exposure, by means of quantitative reverse-transcription polymerase chain reaction (qRT-PCR) and flow cytometry. The immunosuppressant effects of f-LSCs on healthy activated PBMCs were investigated in cell-cell contact and transwell settings through cell cycle assay, acridine orange staining, and caspase-3 detection. We also studied T-cell responses and possible Treg conversion by means of flow cytometry. Functional assays were conducted in activated HT lymphocytes cocultured with f-LSCs after carboxyfluorescein succinimidyl ester labeling and intracellular detection of pro- and anti-inflammatory cytokines.

**Results:**

The hypo-immunogenicity of the f-LSC population depended on both cell contact and soluble factors produced, as well as the undetectable expression of all those molecules required to fully activate T lymphocytes. Following exposure to Th1 cytokines, f-LSCs augmented expression of programmed death-ligand 1 and 2 (PDL-1 and -2), indoleamine-pyrrole-2,3-dioxygenase (IDO), interleukin (IL)-6, and monocyte chemotactic protein 1 (MCP-1) while maintaining their negative phenotype for major histocompatibility (MHC) class II and costimulatory molecules. During coculture, f-LSCs suppressed up to 40% of proliferation in healthy activated PBMCs, arrested them in the G0/G1 cell cycle phase without inducing apoptosis cascade, inverted the CD4/CD8 ratio, and promoted sustained expression of the immunomodulator marker CD69. Under coculture conditions the Th imbalance of autoreactive T cells from female HT patients was fully restored.

**Conclusions:**

Our study describes an in vitro coculture system able to prevent inappropriate activation of autoreactive T lymphocytes of female HT patients and to generate a tolerogenic environment even in an inflammatory background. Further investigations are necessary to establish whether this stem cell-based therapy approach in HT could avoid lifetime hormone replacement therapy by inducing T-cell education.

**Electronic supplementary material:**

The online version of this article (doi:10.1186/s13287-017-0611-5) contains supplementary material, which is available to authorized users.

## Background

It has been proposed that mesenchymal stem cells (MSCs) can contribute to the control of inflammatory diseases, as has been demonstrated by the MSC-mediated attenuation of inflammation in myocarditis [1], rheumatoid arthritis [2], and experimental autoimmune diseases [3, 4]. Due to their regenerative and immunosuppressive properties, MSCs derived from different adult tissue have become a preferred cell type in the field of regenerative medicine and immunotherapy. Although bone marrow is considered a universal source of multipotent MSCs (BM-MSCs), displaying the greatest suppressive effects on T-cell proliferation, the invasive procedure necessary to harvest these cells, the risks of complications, and the age-dependent decline of their self-renewal capacity have led to a search for alternate sources of MSCs. Cord blood-derived MSCs (CB-MSCs), placenta-derived MSCs (P-MSCs), and adipose-derived MSCs (A-MSCs) have been suggested as alternative sources of MSCs for experimental and clinical purposes since they are free from ethical concerns, easy to procure, and available in large quantities. Despite this, only BM- and A-MSCs have the trilineage differentiation potential satisfying the minimal criteria for an MSC as defined by the International Society for Cellular Therapy [5]. Furthermore BM-, CB-, and A-MSCs exhibited replicative senescence when they reached passage 10 on average, whereas P-MSCs expanded until passage 15 [6]. An attempt at a stem cell educator therapy induced by CB-MSCs has already been described [7–9], but only in type 1/2 diabetes and alopecia areata. Limitations for the use of CB-MSCs include insufficient quantity and quality of MSCs obtainable from a single unit of CB, the potential for transfer of genetically abnormal or premalignant cells in patients, and the lack of a National Cord Blood Policy able to manage daily operations of cord blood banking and allocation nationwide.

Nowadays, increasing attention is focusing on application of limbus-derived cells in regenerative medicine. This area of the eye is placed in the junction of the cornea and conjunctiva. It is extensively used for ocular surface resurfacing in patients with limbal stem cell deficiency (LSCD). The cornea is devoid of blood vessels and is assumed to give protection against immune rejection of transplanted grafts, a condition termed “corneal immune privilege” [10]. Its avascularity implies a lack of angiogenic factors or the possibility that it may secrete antiangiogenic factors. In addition, the absence of corneal lymphatic vessels prevents channeling of antigen-presenting cells to the regional lymph nodes. Moreover, immune tolerance in the anterior chamber is responsible for the success rate of corneal transplantation [11]. In this connection, the limbus is a highly specialized region of the eye hosting a well-recognized population of limbal epithelial stem cells (LESCs), which continuously renew the corneal surface [12]. There is more recent evidence that the limbal niche also hosts stromal fibroblast-like stem cells (f-LSCs) with multilineage transdifferentiation potential [13]. We recently demonstrated that f-LSCs represent a robust source of adult stem cells with differentiation potential towards the pancreatic endocrine cell fate, good proliferative capability, and long-term maintenance of stem cell properties independently of donor age and long-term culture conditions [14, 15].

Autoimmune thyroid disease (AITD) is the most common thyroid disease. These organ-specific autoimmune disorders include intrathyroidal lymphocyte infiltration, presence of circulating thyroid autoantibodies, immunological overlap with other autoimmune diseases, and a history of familial occurrence, mainly in females. It occurs due to loss of tolerance to thyroid autoantigens such as thyroperoxidase (TPO), thyroglobulin (Tg), and thyroid stimulating hormone receptor (TSH-R) leading to T- and B-cell infiltration into the gland [16]. The destruction of thyroid parenchyma in Hasimoto’s thyroiditis (HT) is mostly due to gland infiltration by cytotoxic T cells, production of autoantibodies, and cytokine-mediated induction of Fas on thyrocytes, which constitutively express FasL [17]. Various cytokines are found in thyroid follicular cells which enhance inflammatory response with nitric oxide (NO) and prostaglandins [18]. In particular, interferon (IFN)-γ is a proinflammatory cytokine that induces expression of human leukocyte antigen class II (HLA-DR) molecules in many cell types including BM-MSCs. We showed that f-LSCs, unlike other kinds of MSCs, do not modify their immuno-privileged condition, even if an inflammatory background exists, acting as “smart immunomodulators”. Ultimately their in vitro immunosuppressant capability offers a novel concept of stem cell-based therapy for the treatment of a complex disease such as HT with unknown pathogenesis and currently monitored through a symptomatic therapy merely based on administration of synthetic thyroid hormone. Our aim was to evaluate the ability of f-LSCs to exert immunomodulation on peripheral blood mononuclear cells (PBMCs) from female HT patients studying different mechanisms.

## Methods

### Isolation of limbal stem cells

Human corneo-scleral rings from donors were processed as previously described [14]. The study was approved by the Ethical Committee of the AOUP, University of Palermo (No. 09/2009).

Briefly, samples underwent fine dissection with a sterile blade and were subsequently incubated with collagenase I (5 mg/ml; Sigma-Aldrich, St. Louis, MO, USA) overnight at 37 °C in a shaking bath and the day after placed in p60 culture dishes (Corning, New York, USA) with the fibroblast maintenance medium (Dulbecco’s modified Eagle’s medium (DMEM)/F12 supplemented with 10% embryonic stem cell-tested fetal bovine serum (EC-FBS; PAA Laboratories Gmbh, Austria), 1× 5 μg/ml insulin, 5 μg/ml transferrin, and 5 μg/ml selenium (ITS; PAA Laboratories), and 20 ng/ml basic fibroblast growth factor (b-FGF; Preprotech, London, UK) until cells reached confluence. The f-LSC subcultures were kept in the expansion medium (DMEM/F12 supplemented with 5% EC-FBS (PAA Laboratories), 1× ITS (PAA Laboratories), and 4 ng/ml b-FGF (Preprotech)) up to passage 20.

### Patient selection

Thirty-one female patients aged between 28 and 66 years with autoimmune thyroiditis and elevated plasma TPO antibodies (TPOAb) and/or Tg antibodies (TgAb) were selected at the Outpatient Clinic of the Section of Endocrinology, Policlinico P. Giaccone, University of Palermo, and asked for their informed consent to participate in the study. Diagnoses had been made using elevated TPOAb/TgAb. All patients were receiving L-T4 replacement therapy in a dosage to maintain basal TSH within the normal range. Patients with a history of allergies, other chronic diseases (e.g., diabetes, hypertension, coronary heart, viral hepatitis), or receiving corticoids or other anti-inflammatory therapies were excluded from the study. The healthy control group comprised 18 subjects: 12 males and 6 females aged 24–34 years. Fifteen milliliters of heparin anticoagulated blood was drawn from each donor/patient in the morning after a 12-h fasting period. The blood was diluted 1:1 with phosphate-buffered aline (PBS) solution, and PBMCs were separated by gradient centrifugation over Ficoll (Lympholyte-Human Cell Separation Media, Cedarlane, Burlington, Canada) according to the manufacturer’s instructions.

### Isolation of T cells

For some experiments untouched T cells were purified using the Human Pan T Cell Isolation Kit (Miltenyi Biotec, Bergisch Gladbach, Germany). The purity of isolated cells was >95% as assessed by flow-cytometric analysis. Viability (>95%) was determined by Trypan blue exclusion.

### Cocultivation experiments

PBMCs obtained by Ficoll-Paque density gradient or purified T cells were cultured in complete RPMI-1640 (PAA Laboratories) supplemented with penicillin-streptomycin solution (SigmAldrich, Milan, Italy) and 10% heat-inactivated fetal bovine serum (bovine serum albumin (BSA); PAA Laboratories). In coculture experiments the PBMCs were activated for 72 h with 5 μg/ml of mAb anti-human CD3 (OKT-3 Clone, SigmAldrich). The T cell purified culture was supplied with the OKT-3 Clone mAbs along with 1 μg/ml of anti-human CD28 mAbs (SigmAldrich) for the same time period. f-LSCs were seeded at 2000 cells/cm^2^ and allowed to adhere in 24-well or 6-well plates (Corning Co., Corning, NY) overnight. After 24 h they were cocultured with total PBMCs or purified T cells at a 1:50 or 1:100 ratio, respectively. Preliminary experiments were performed to establish the optimal cell ratio by varying the f-LSCs concentration. The low f-LSC/PBMC ratio in the flasks prevented f-LSC detachment while the PBMCs or T cells were gently recovered as supernatant for FACS and quantitative reverse transcription polymerase chain reaction (qRT-PCR) analyses. For some experiments the stimulated PBMCs were directly plated in 0.4-μm Transwell inserts (Corning Co.) and put in a 24 well-plate in which f-LSCs were precultured for 72 h. PBMCs/T cells without TCR stimulation were used as negative controls. Cytokine prelicensing of f-LSCs was performed by adding 500 U/ml of recombinant human IFN-γ, interleukin (IL)-1β, and IL-6 (all by Peprotech, Rocky Hill, NJ, USA) for 48 h. In all coculture experiments BM-MSCs were used as internal positive controls of stemness. The best PBMC/BM-MSC ratio used was 1:10, as reported in many previous in vitro studies [19, 20].

### Isolation of total RNA and qRT-PCR

Total RNA was extracted and purified from PBMCs or f-LSCs using the RNeasy Mini Kit (Qiagen, Milan, Italy), including a digestion step with DNase I step according to the manufacturer’s protocol. RNA quantity and quality were assessed by means of UV spectrophotometry; 1 μg total RNA were reverse transcribed in a volume of 20 ml with Oligo dT primers (Applied Biosystems, Darmstad, Germany) and Stratascript RT (Stratagene, Amsterdam, Netherland), according to the manufacturer’s protocol. The primer pair sequences are listed in Table 1. The primer sets for HLA-G were selected to amplify all alternative forms of HLA-G transcripts. PCR primers for IL-6, FAS, IDO, MCP1, CCND1, and p27 were purchased from Qiagen (QuantiTect Primer Assays, Qiagen, Milan, Italy). All reactions were performed with Quantitect Sybr Green PCR Kit (Qiagen) using the Rotor-Gene Q instrument (Qiagen). The specificity of the amplified products was determined by means of melting peak analysis. Relative gene expression analysis for each gene was performed with Rotor-Gene Q software using the Delta Delta Ct method validated according to the guidelines of Livak and Schmittgen [21]. All reactions were performed at least in triplicate.

**Table 1:** Primer sequences used for RT-PCR analysis

Gene	Primer sequence
PDL1 (CD274)	Forward primer 5′ TTGCTGAACGCCCCATACAA 3′ Reverse primer 5′ GGAATTGGTGGTGGTGGTCT 3′
TGF β1	Forward primer 5′ GTGGACATCAACGGGTTCACT 3′ Reverse primer 5′ ATGAGAAGCAGGAAAGGCCG 3′
FasL	Forward primer 5′ GCAGCCCTTCAATTACCCAT 3′, Reverse primer 5′ CAGAGGTTGGACAGGGAAGAA 3′
AIRE	Forward primer 5′ CGGGGGTATAACAGCGGC 3′ Reverse primer 5′ CCTCAGAAGCCGGCGTAG 3′
HLA-G	Forward primer 5′CTGGTTGTCCTTGCAGCTGTAG 3′ Reverse primer 5′ CCTTTTCAATCTGAGCTCTTCTTTCT 3′
COX2	Forward primer 5′ ATCATTCACCAGGCAAATTGC 3′, Reverse primer 5′ GGCTTCAGCATAAAGCGTTTG 3′
HGF	Forward primer 5′ CTC ACA CCC GCT GGG AGT AC 3′ Reverse primer 5′ TCC TTG ACC TTG GAT GCA TTC 3′

*AIRE* autoimmune regulator, *COX2* cyclooxygenase-2, *HGF* hepatocyte growth factor, *HLA-G* human leukocyte antigen G, *PDL1* programmed death-ligand 1, *RT-PCR* reverse-transcription polymerase chain reaction, *TGF* transforming growth factor

### Cell cycle assays

Cell cycles were performed according to the protocol of Nicoletti et al. [22] and analyzed by flow cytometry (FACSCalibur, Becton Dickinson). Briefly, cell suspensions were fixed in 70% ethanol and stained with propidium iodide (PI) overnight before FACS analysis.

### Acridine orange/ethidium bromide staining

PBMC suspensions (10^6^) after 72 h of coculture were incubated with 1 μl acridine orange/ethidium bromide (AO/EB) solution (5 mg/ml and 3 mg/ml, respectively, in PBS) and mixed gently. Then 10 μl of each stained sample was placed onto a microscopic slide cover with a glass coverslip and immediately evaluated under a fluorescence microscope using a fluorescein filter in a 40× objective. Jurkat cells treated for 4 or 24 h with human activating anti-Fas antibody (CH11 clone) were used as apoptotic positive controls.

AO/EB staining was also performed in f-LSCs. They were cultured in chamber slides (BD Biosciences) for 48 h with or without Th1 cytokines (IL-1β, IL-6, IFN-γ) and afterwards stained as described above.

### Flow cytometry

The cells were treated with FcR blocking reagent (Miltenyi Biotec, Bergisch Gladbach, Germany) and incubated with each fluorochrome-conjugated antibody or appropriate isotype control at 4 °C for 30 min in the dark. Cells were then fixed for 15 min at 4 °C with 2% paraformaldehyde (PFA) and washed with staining buffer (PBS, calcium and magnesium free, supplemented with 1% BSA (Sigma-Aldrich)). The T-cell phenotype was determined using CD25 PerCP-Cy™5.5, FoxP3 (Scurfin, IPEX, JM2) PE, CD4 FITC, CD69 PE, CD8 FITC/PE, CD3 FITC, CD152 (CTLA-4) PE, CD28 (TLR2) PE, IFN-γ PE, IL-4 PE, IL-17 PE, RORγt PE, and IL-10 PE (all purchased from BD Biosciences, Milan, Italy). Intracellular staining was performed using BD Cytofix/Cytoperm™ Plus Fixation/Permeabilization Kit (with BD GolgiStop™ protein transport inhibitor) (BD Biosciences, Milan, Italy) according to the manufacturer’s instructions. For cytokine detection, BD GolgiStop protein transport inhibitor containing monensin was added to the culture for 5 h before cell harvesting.

The f-LSC immunophenotype was determined using the following monoclonal antibodies: HLA-DR FITC, CD80 (B7-1) PE, CD86 (B70/B7-2) PE, PD-1 (CD279) PE, CD34 FITC, CD45 FITC, CD274 (B7-H1, PD-L1) PE, CD273 (B7-DC, PD-L2) PE, and B7-H4 PE (BD Biosciences). Freshly isolated PBMCs and primary CD34^+^ BM-MSCs (Lonza, Basel, Switzerland; catalogue number 2 M-101C) were used as positive controls for hematopoietic and stem cell/immunosuppressive markers, respectively. For apoptosis detection, active caspase-3 antibody, reported to specifically recognize the active form of caspase-3 in humans, was used (BD Biosciences). All data were acquired on a FACSCalibur and analyzed using CELLQuest Pro software (BD Pharmingen, San Jose, CA, USA).

### MTT assay

Cell proliferation was assessed by colorimetric assay using 3-(4,5-dimethylthiazol-2-yl)-2,5 diphenyltetrazolium bromide (MTT) according to Mosmann’s protocol [23]. f-LSCs with or without cytokines were plated in a 96-well plate with 100 mL medium/well and cultured for up to 72 h. The proliferation rate was evaluated by UV absorption spectrum at 550 nm, after MTT incubation for 4 h at 37 °C. 

### Proliferation assay

PBMCs from healthy donors or female HT patients were labeled with CellTrace carboxyfluorescein succinimidyl ester (CFSE) using the Cell Proliferation Kit (Molecular Probes Invitrogen, Milan, Italy), according to the manufacturer’s instructions. Labeled PBMCs were resuspended in RPMI 1640 medium with 10% FBS, activated with 5 μg/mL of anti-CD3 mAbs and cocultured with 1-day plated f-LSCs. After 7 days of coculture PBMCs were gently harvested from the supernatant and CFSE fluorescence detected by flow cytometry. Samples were analyzed by Modfit LT Version 3.2 software (Verity Software House) and the proliferation index calculated as the sum of cells in all generations divided by the number of original parent cells.

### Statistical analysis

All assays were performed in triplicate. The data are reported as means ± SD and compared using the appropriate version of the Student’s unpaired *t* test. Test results were reported as two-tailed *p* values, where *p* < 0.05 was considered statistically significant.

## Results

### Immunophenotype of f-LSCs

**Fig. 1:**
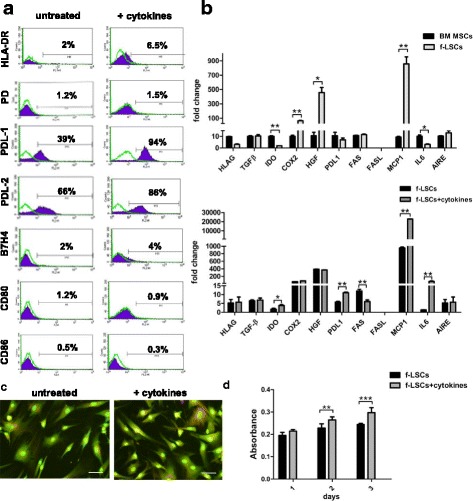
Immunophenotyping of f-LSCs. **a** The immunological profile of fibroblast-like limbal stem cells (*f-LSCs*) was assessed by means of flow cytometry before (*left panel*) and after (*right panel*) 48 h of cytokine-treatment (IL-1β, IL-6, and IFNγ, 500 U/ml) for the human leukocyte antigens (*HLA-DR*), the immune T-activator molecule programmed death-1 (*PD-1*, CD279) and its ligands (programmed death-ligand 1/2 (*PDL-1/2*)), the negative regulator of T cell response B7-H4 and the costimulatory molecules CD80 (B7-1) and CD86 (B70/B7-2). All histogram plots include the percentage of expression of each protein as a representative value of five independent experiments. **b** qRT-PCR shows the expression of several immunosuppressive and tolerogenic markers (HLA-G, transforming growth factor beta (*TGF-β*), indoleamine-pyrrole 2,3-dioxygenase (*IDO*), cyclooxygenase-2 (*COX-2*), hepatocyte growth factor (*HGF*), PDL-1/2, Fas, Fas-L, monocyte chemotactic protein 1 (*MCP-1*), interleukin-6 (*IL-6*), and autoimmune regulator (*AIRE*)) compared to the bone marrow-derived mesenchymal stem cell (*BM-MSC*) positive control (*upper panel*). The same markers were tested in f-LSCs after cytokine stimulation. The fold change for each gene was calculated with the Delta Delta Ct method using the Rotor Gene Q software and the expression was normalized for the housekeeping gene β-actin. **c** f-LSCs were cultured with the abovementioned Th1 cytokines for 48 h in T-25 flasks. After acutase-mediated dissociation, 10^6^ cells were used for AO/EB staining. Pictures show the viability of untreated and cytokine treated cells using a fluorescein filter in a 40× objective. **d** Proliferation assay (MTT) at 48 h for untreated and cytokine-treated f-LSCs. All data/pictures are representative of at least five independent experiments. Values on the bars are shown as mean ± SE; *p < 0.05, **p < 0.02, ***p < 0.01

f-LSCs at a relatively early passage (passage 1–10) exhibited the expected fibroblast-like morphology and expressed the phenotypic markers shown in Fig. 1a (left panel) and 1b (upper graph). qRT-PCR and flow cytometry analysis were performed to determine the presence of key factors involved in immunoregulation. Up to passage 20 over 95% of growing cells were negative for the costimulatory molecules CD80 (B7-1), CD86 (B70/B7-2), HLA-DR, the immune T-activator molecule programmed death-1 (PD-1, CD279), the hematopoietic markers CD34 and CD45, and the epithelial marker Δp63. By contrast, the majority of them expressed the stemness markers SSEA4, SOX2, CD105, OCT4, NANOG, and CD90 and were able to differentiate into adipocytes, chondrocytes, osteocytes and insulin-producing cells as previously published [13, 14]. It was found that f-LSCs constitutively expressed, and at the same levels as BM-MSCs, transcripts required to modulate an immune response such as transforming growth factor (TGF)-β, programmed death-ligand 1 (PDL-1), the autoimmune regulator (AIRE), and FAS (CD95). The weak expression for CD95 and the total absence of expression for CD95 ligand (CD95L) in f-LSCs did not suggest a Fas-FasL mediated immunoregulation. In contrast, constitutive HLA-G, indoleamine-pyrrole 2,3-dioxygenase (IDO), and IL-6 expression were approximately four- to fivefold lower compared to the positive controls. The primer set used in this study detected both the HLA-G membrane bound (HLA-G1, G2, G3, and G4) and soluble isoforms (HLA-G5, G6, and G7) [24]. Interestingly, expression of hepatocyte growth factor (HGF), cyclooxygenase-2 (COX-2), and monocyte chemotactic protein-1 (MCP-1 or CCL2) was found to be 10-, 50-, and 90-fold higher, respectively, in f-LSCs compared to BM-MSCs. In addition, f-LSCs showed an appreciably constitutive expression for CD274 (PDL-1) and CD273 (PDL-2) (39.0 ± 5.1%; 66.0 ± 3.4%) and the absence of the negative regulator of T cell response B7-H4 (2.1 ± 0.5%) at the protein level. Taken together, these findings suggested that the f-LSCs exhibited the machinery required to induce immunomodulation and immunosuppression.

### Proinflammatory ‘licensing’ of f-LSCs

In contrast to therapies that cause global immune suppression, some MSCs have been dubbed as “smart immune modulators” since their suppressive effects require a previous licensing step that occurs in the presence of an inflammatory milieu and is mediated by the secretion of specific cytokines [25]. To explore this phenomenon in f-LSCs we cultured them with several Th1-related cytokines (IL-1β, IL-6, and IFN-γ at 500 U/ml) for 48 h miming in vitro the inflammatory environment provided from lymphocytes in HT. Next we assessed their immunophenotyping by qRT-PCR and flow cytometry. FACS analysis showed an increase in PDL-1 and PDL-2 protein expression (40.5 ± 2.3% vs. 91.0 ± 3.5% and 66.8 ± 4.2% vs. 86.5 ± 2.5%, respectively; *p* < 0.02) and no significant changes for CD80, CD86, B7H4, PD-1, and HLA-DR (Fig. 1a, right panel). Our results also showed maintenance at baseline of mRNA for HLA-G, TGF-β, and AIRE. Expression of COX-2 and HGF was preserved at sustained levels without significant variations. The apoptotic marker CD95 halved in expression while CD95L was still totally absent. Notably, mRNA for IDO and PDL-1 was found to be upregulated twofold compared to untreated controls while the mRNA for IL-6 and MCP-1 increased 25- and 60-fold, respectively (Fig. 1b, lower graph). During stimulation f-LSCs appeared to show regular morphology when compared to the untreated negative controls (Fig. 1c) and to grow moderately faster, as shown by the MTT assay (Fig. 1d). Taken together, these data confirmed the capability of f-LSCs to enhance their immunosuppressive phenotype increasing the key immunomodulator markers PDL-1, PDL-2, IDO, IL-6, and MCP1 in an inflammatory environment. Furthermore, this trend did not affect the expression of the costimulatory markers CD80 and CD86 and the immune regulator molecule PD-1. In particular we observed minimal positivization of the major histocompatibility (MHC) class II cell surface receptor unlike the cytokine stimulated BM-MSCs used as internal controls of stemness (5.8 ± 1.0% vs. 60.4 ± 4.5%; *p* <0.01) (Additional file 1A). These data clearly suggest that f-LSCs are superior immunomodulators with respect to BM-MSCs and have minimal immunogenicity even in an inflammatory environment.

### Inhibitory effect of f-LSCs on TCR-triggering activated PBMCs from healthy donors and after mixed leukocyte reactions

**Fig. 2:**
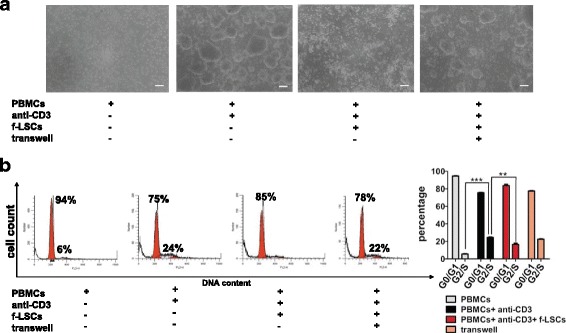
Inhibitory effect of f-LSCs on PBMC proliferation. **a** Peripheral blood mononuclear cells (*PBMCs*) from healthy volunteers were stimulated with anti-CD3 for 72 h in the presence or absence of fibroblast-like limbal stem cells (*f-LSCs*) (1:50 ratio). The f-LSCs drastically reduced the clump formation after coculture. In the transwell system the lymphomonocyte density was slightly lower than activated PBMCs alone, used as positive controls. No activated PBMCs served as negative controls. Original magnification: 40×. Pictures are representative of at least five independent experiments. **b** DNA content was assessed using PI staining and analyzed by flow cytometry. FACS plots are representative of five experiments of identical design. Values on the bars are shown as mean ± SE; **p < 0.02, ***p < 0.01

f-LSCs were cocultured with anti-CD3 stimulated PBMCs from healthy volunteers at a ratio of 1:50 for 72 h. Using phase-contrast microscopy we observed that activated lymphocytes formed countless quantities of cell clumps of different sizes in the absence of f-LSCs. However, the number of cell clumps was significantly reduced after f-LSC coculture and the majority of lymphocytes were individually distributed in the medium or closely adhered to the f-LSCs. In the transwell system the lymphomonocyte density and blastization rate was slightly lower than that of the activated PBMCs alone (Fig. 2a). Comparable results were obtained in mixed leukocyte reactions with the addition of f-LSCs (Additional file 2A).

### f-LSCs cause cell cycle arrest of activated PBMCs from healthy donors

MSCs were found to arrest T cells in the G0/G1 phase of the cell cycle. This form of T-cell unresponsiveness, which is triggered by MSCs, is not the classical form of anergy but is referred to as “tolerance arrest” in T cells [26]. To explore this effect, DNA content was measured using PI staining in activated PBMCs from healthy donors under different coculture conditions. Analysis of the cell cycle showed that 22.4 ± 5.3% of PBMCs after 72 h of activation were in the G2/S phase. In the presence of f-LSCs, most cells (85.0 ± 1.2%) remained in the G0/G1 phase, almost like the unstimulated control cells (94.0 ± 2.8%), while in the transwell system no significant inhibition of PBMC proliferation was observed (20.2 ± 3%) (Fig. 2b). A previous study on T-cell cycle entry defined a commitment point at early G1 where cells decide whether to enter the cell cycle; it is associated with the induction of cyclin D1 expression [27]. To identify the specific point at which T-cell proliferation was arrested by f-LSCs we evaluated the expression of the principal molecules involved in cell cycle regulation. For this purpose, PBMCs from healthy volunteers were stimulated with anti-CD3 in the presence or absence of f-LSCs and evaluated by qRT-PCR for expression of cyclin D1 and p27Kip1 as G1 phase-specific markers. Unstimulated PBMCs with or without f-LSCs exhibited constitutive levels of cyclin D1 and a high expression level of the negative cell cycle regulatory protein p27Kip1. In response to anti-CD3, cyclin D1 did not change in expression whereas p27Kip1 was significantly downregulated to induce cell cycle entry. Notably, the expression of p27Kip1 was induced in stimulated PBMCs that had been cocultured with f-LSCs suggesting the possibility that f-LSCs exerted their growth inhibitory effect primarily through induction of p27Kip1 expression (Additional file 2B). For all coculture experiments human BM-MSCs served as internal positive controls, revealing a capacity for inhibition of proliferation 10% higher than that of f-LSCs in healthy activated PBMCs after coculture (Additional file 3A and B).

### T-cell inhibition by f-LSCs is not due to induction of apoptosis

**Fig. 3:**
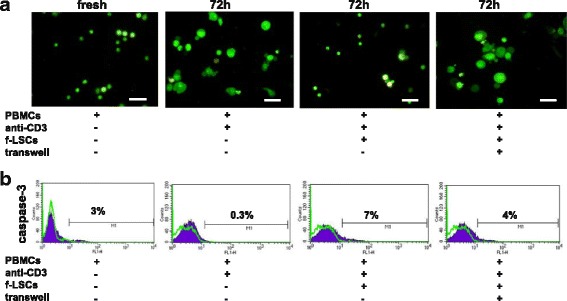
The immunosuppressive effects of f-LSCs is not mediated by apoptotic mechanisms. **a** AO/EB staining was performed as described in the Methods section on healthy PBMCs. The experiments included the following conditions: fresh unstimulated peripheral blood mononuclear cells (*PBMCs*), activated for 72 h with anti-CD3 mAbs, after coculture with fibroblast-like limbal stem cells (*f-LSCs*), and in the transwell system for the same time lapse. No typical signs of apoptotic event were observed under all four conditions. Original magnification: 40×. All pictures are representative of five independent experiments. **b** Active caspase-3 was detected by FACS showing a very faint involvement of apoptotic cascade in activated healthy PBMCs after 72 h of coculture with f-LSCs. FACS plots are representative of five experiments of identical design

To test whether or not the inhibitory effect of f-LSCs was associated with the apoptotic event, AO/EB staining was concurrently assessed with active caspase-3 detection by flow cytometry. AO/EB was used to distinguish between quiescent, activated, and proliferating cells and to measure apoptosis. After 72 h of culture, PBMCs stained orange and started to show marks of necrotic death, as indicated by ethidium bromide intercalation. After 72 h activated PBMCs appeared uniformly green, bigger, and with a bulky cytoplasm. In coculture with f-LSCs the same cells appeared fewer in number, not apoptotic or necrotic, but still moderately activated (Fig. 3a). Jurkat cells treated for 4 or 24 h with human activating anti-Fas (CH11 clone) were used as apoptotic positive controls (Additional file 4A) showing many apoptotic body formations and bright green dots in the nuclei as a consequence of chromatin condensation and nuclear fragmentation. Late apoptotic cells were observed with condensed and fragmented nuclei, especially after 24 h of anti-Fas treatment. Confirming these observations, the percentage of active caspase-3-positive cells in the presence of f-LSCs and under transwell conditions showed 6.0 ± 2.8% and 4.3 ± 0.5% of positivity, respectively. Overall, these data suggest that f-LSCs inhibit the proliferation of PBMCs mainly by nonapoptotic mechanisms. The weak involvement of apoptosis in immunosuppression of lymphocytes induced by BM-MSCs is shown in Additional file 5A and has been confirmed in the past by others [28, 29].

### Regulation of f-LSCs on conventional activation markers in the CD3^+^ subset, CD4^+^/CD8^+^ ratio, and CD4^+^CD25^+^ regulatory T lymphocytes

**Fig. 4:**
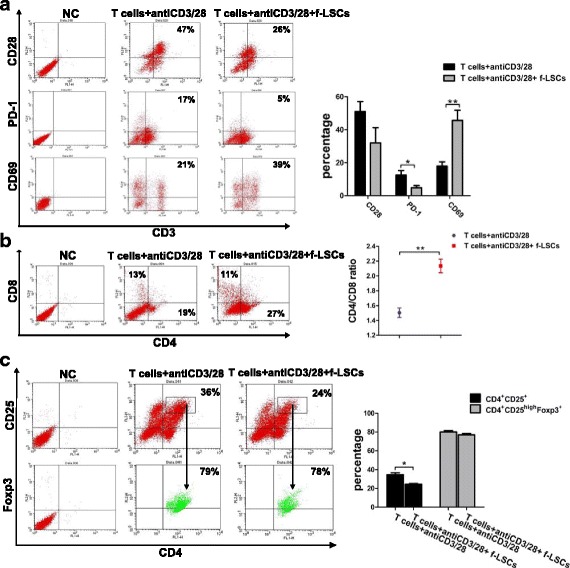
f-LSCs negatively regulate the proliferation of CD4^+^, CD8^+^ lymphocytes and the induction of CD4^+^CD25^+^ regulatory T cells. **a** Representative dot plots of activated purified T cells collected by healthy volunteers and stained for the three activation markers CD28, programmed death (*PD-1*), and CD69 (*left panel*) are shown. Samples were run after 72 h of incubation with or without fibroblast-like limbal stem cells (*f-LSCs*). **b** A representative flow staining of activated CD4^+^ and CD8^+^ lymphocytes alone or after coculture is shown (*left panel*). In the right section the consequential CD4/CD8 ratio was calculated. **c** Gating strategy used to detect the CD4^+^CD25^high^Foxp3^+^ fraction in T cells collected by healthy volunteers and stimulated with anti-CD3/CD28 for 3 days with or without f-LSCs. In all experiments unstimulated PBMCs were used as a negative control. Numbers in the corner of each quadrant correspond to the percentage of the corresponding cell population inside any dot plot. Also shown are the average results from five independent experiments. Data are presented as means ± SE in each histogram; *p < 0.05, **p < 0.02. *NC* normal control

To evaluate immune regulation of f-LSCs on T subsets, f-LSCs were cocultured for 72 h with purified T cells from healthy volunteers in the presence of anti-CD3/28 mAbs and analyzed by flow cytometry for some conventional activation molecules (CD28, PD-1, CD69) and for CD4 and CD8 T-surface markers. After coculture we found marked downregulation of the two activation markers CD28 and PD-1 in T cells collected from young volunteers (50.5 ± 3.3% vs. 25.8 ± 3.2% and 17.0 ± 1.0% vs*.* 5.1 ± 1.6%, respectively). Higher levels of CD69 (21.0 ± 1.9% vs*.* 39 ± 4.0%) were detected under the same conditions (Fig. 4a) inside the CD3^+^ T-cell subset. Furthermore, after f-LSC stimulation the percentages of total CD4^+^ increased (19.3 ± 0.8% vs. 27.3 ± 0.2%) whereas the CD8^+^ fraction level was similar to the control (13.1 ± 0.5% vs. 11.0 ± 0.2%). Ultimately, the CD4/CD8 ratio was appreciably upregulated (Fig. 4b). This result suggested that f-LSCs may display regulation on the activation status of T-cell compartments and on CD4^+^ and CD8^+^ T-cell subsets. CD28, PD-1, and CD69 expression was found to be downregulated on activated CD3^+^ T cells upon stimulation with BM-MSCs for 72 h, confirming the different nature of the two stem cell populations and mechanism of action in immunomodulation induction. A similar trend in the inversion of the CD4/CD8 ratio inside the purified pool of T cells after coculture with BM-MSCs was found (Additional file 5B and C). We next investigated whether the inhibitory effects of f-LSCs involved expansion of Treg cells, which are CD4^+^CD25^high^Foxp3^+^ T cells capable of modulating tolerance in immune response [30]. We performed flow cytometric analysis for CD4^+^CD25^+^ cells from T cells of healthy controls after 3 days of incubation with f-LSCs in the presence of anti-CD3/28 mAbs. We found a faint reduction in the CD4^+^CD25^+^ fraction among stimulated lymphocytes as shown in Fig. 4c. This could be the consequence of a lower activation regimen induced by f-LSCs on T cells. Notably, inside the gated CD4^+^CD25^high^ population no differences in percentage of CD4^+^Foxp3^+^ cells were detected when f-LSCs were added to the culture. As expected, and as previously proved by others, the CD4^+^Foxp3^+^ regulatory T cells were minimally expanded from BM-MSCs after coculture (82 ± 5.9% vs. 94 ± 3.2%) (Additional file 5D) [31–33].

Taken together, these results indicated that f-LSCs displayed immune regulation on both CD4^+^ and CD8^+^ T-cell subsets.

### Comparison of inhibitory effects induced by f-LSCs in PBMCs from female HT patients and healthy donors

**Fig. 5:**
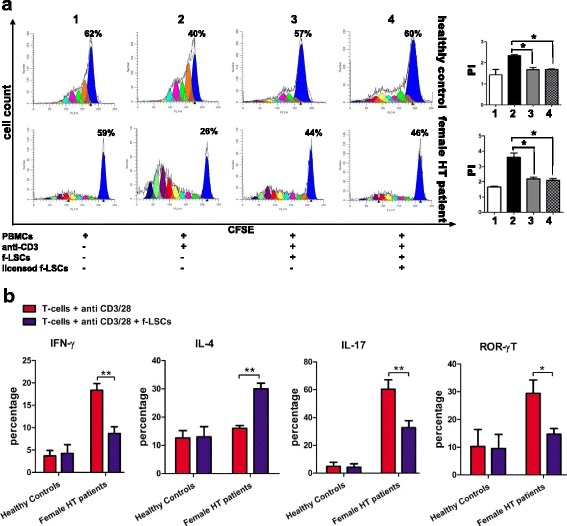
Immunomodulatory effects induced by f-LSCs in PBMCs/T cells harvested from female HT patients. **a** Peripheral blood mononuclear cell (*PBMC*) proliferation was assessed by the CFSE method after 7 days of coculture and analyzed with FACS. The proliferation index (*PI*) correlated with the number of cell divisions that PBMCs had undergone. **b** A comparison of FASC analysis for interferon gamma (*IFN-γ*), interleukin (*IL*)-4, IL-17A, and ROR-γT expression in activated purified T cells collected from female Hashimoto’s thyroiditis (*HT*) patients and healthy donors after 3 days with or without fibroblast-like limbal stem cell (*f-LSC*) stimulation. Data are derived from one of the five independent experiments and are presented, inside the histogram, as means ± SE; *p < 0.05, **p < 0.02

Next, using CFSE, we compared the proliferation rate of responding PBMCs from healthy controls and female HT patients stimulated with anti-CD3 mAbs with or without f-LSCs for 72 h. Unstimulated PBMCs without f-LSCs were used as negative controls. The proliferation index, useful for determining the antiproliferative effects of f-LSCs on activated lymphocytes, was calculated as the sum of cells in all generations divided by the number of original parent cells. The CFSE results are shown in Fig. 5a and were similar to those obtained using manual counting. Specifically, PBMCs from healthy volunteers and female patients, grown in culture without mitogen antibodies, after a 7-day period of incubation yielded a proliferation index of 1.44 ± 0.08 and 1.43 ± 0.1, respectively (negative controls). Antibody stimulation significantly increased the proliferation of lymphocytes (proliferation index 2.5 ± 0.1 in healthy controls vs. 3.84 ± 0.5 in female patients). The coculture of stimulated PBMCs with f-LSCs produced a proliferation index of 1.75 ± 0.1 in volunteers vs. 2.27 ± 0.5 in female patients, representing a significant impairment of PBMC activation that was slightly more marked in lymphocytes from patients compared to healthy controls. The 48 h cytokine prelicensing of f-LSCs with IL6, IL-1β, and IFN-γ did not significantly improve their immunosuppressive performance, reducing the lymphocyte-blastization rate approximately to the same levels as untouched f-LSCs (Fig. 5a).

### f-LSCs downmodulate in vitro production of inflammatory cytokines

Several studies have suggested that MSCs modulate the differentiation, function, and balance of the Th1, Th2, and Th17 subpopulations and foster development of an anti-inflammatory immune response [34]. To identify factors that could be involved in immunosuppressive effects mediated by f-LSCs, production of proinflammatory or immunosuppressive cytokines and expression of the transcription factors ROR-γT were investigated by means of intracellular flow staining. The results showed that, in activated T cells from female HT patients, intracellular production of IFN-γ, IL-17A, and ROR-γT was considerably decreased in cocultured cells compared to untreated controls (18.0 ± 2.1% vs. 9.0 ± 1.9%; 35.2 ± 6.5% vs. 18.4 ± 3.4%; 27.0 ± 4.5% vs. 14.0 ± 2.6%, respectively). In contrast, at the same levels f-LSCs did not inhibit expression of the aforementioned cytokines on T cells from healthy controls (5.1 ± 1.4% vs. 4.4% ± 1.9%; 8.8 ± 1.2% vs. 7.0 ± 3.4%; 12.5 ± 6.5% vs. 11.0 ± 5.6%, respectively). Notably, IL-4 secretion by Th2 was induced in the presence of f-LSCs, especially in lymphocytes from female patients (15.0 ± 2.1% vs. 28.5 ± 3.9%) compared to healthy donors (15.0 ± 2.6% vs. 17.5 ± 1.5%) (Fig. 5b). These data suggest that the activation of naive T cells toward a Th1 or Th17 immunophenotype depended on an inflammatory milieu, as in HT-activated T cells, and was attenuated in the presence of f-LSCs. These results show, for the first time, the capability of f-LSCs to downmodulate production of inflammatory cytokines by activated lymphocytes of female HT patients. This action could be due to a superior inflammatory background level present in the T cells collected from patients compared to healthy controls.

## Discussion

Hashimoto’s thyroiditis (HT) or chronic lymphocytic thyroiditis is an autoimmune disease in which the thyroid gland is attacked by a variety of cell- and antibody-mediated immune processes [18]. This condition is the most common cause of hypothyroidism in the world in individuals older than 6 years and is about seven times more frequent in women than in men. To date, no clinical approach has aimed to treat the immunological cause of a chronic disease like HT; it is only possible to control its symptoms and clinical manifestations through lifetime thyroid hormone replacement therapy.

Multiple studies have demonstrated the immunoregulatory properties of MSCs. MSCs profoundly affect the immune response through interactions with the cellular components of the innate (natural killer (NK) cells) and adaptive (dendritic cells, B lymphocytes, and T lymphocytes) immune systems. MSC immunoregulation can occur through cellular contact and/or the secretion of diverse factors [35]. Cells with the characteristics of MSCs represent a heterogeneous population of stem cells that have been isolated from almost every type of tissue stroma sharing their principal immunomodulatory and regenerative properties. In human limbus we recently identified stromal fibroblast-like stem cells (f-LSCs) characterized by robust proliferative capacity, stable expression of several pluripotent stem cell markers, self-renewal ability, and multilineage potential. Here, we show, for the first time, that these cells efficiently inhibit lymphocyte proliferation and modulate cytokine production. FACS and qRT-PCR analysis revealed the hypoimmunogenity of f-LSCs as they did not express the complete pattern of molecules required to fully activate T cells. Specifically, they were negative for the MHC class II and costimulatory (CD80 or CD86) molecules, the hematopoietic markers CD34 and CD45, and the epithelial marker ΔNp63 [13, 14]. Their immune privileged phenotype is also due to both cell contact and the soluble factors produced. Among them we detected the following: TGF-β, PDL-1/2, HLAG, IDO, IL-6, HGF, COX-2, and MCP-1 (CCL2). Most importantly human f-LSCs differed from our positive control (BM-MSCs) for the more elevated expression of HGF, COX-2, and MCP-1. HGF and TGF-1β were the first molecules described in MSC-mediated immune regulation of alloantigen-activated T lymphocytes. Both cytokines can independently diminish alloantigen-activated T-lymphocyte proliferation, although proliferation was partially re-established through blocking antibodies [36]. Prostaglandin E2 (PGE2) is a lipid mediator derived from the conversion of arachidonic acid to prostaglandin through COX1 and COX2 enzyme action. PGE2 has been shown to diminish proliferation, stimulate the secretion of IL-4 and IL-10, and promote CD4^+^CD25^+^Foxp3^+^ [37].

IFN-γ is a proinflammatory cytokine that can induce expression of HLA class II (HLA-DR) molecules in many cell types including BM-MSCs. By contrast, f-LSCs act as “smart immunomodulators”, improving their phenotype and their expression for some immune regulatory mediators after exposure to several Th1 cytokines (IL-6, IL-1β, IFN-γ). Consequently their immunosuppressive potential is expected to be superior in all those autoimmunity diseases, including HT, with a Th1-driven pathogenesis [17]. Furthermore, the natural immune privileged state and hypoimmunogenicity of f-LSCs can revoke the need for HLA matching in case of reinfusion of re-educated lymphomonocytes in patients even if an inflammatory status exists. As a consequence of their sensitivity to inflammation we demonstrated that human f-LSCs adjust their phenotype after 48 h of Th1 cytokine treatment, maintaining, at baseline, mRNA expression for HLA-G, TGF-β, IDO, and AIRE; they preserve at sustained levels the mRNA for COX-2 and HGF, upregulate the mRNA for MCP-1 and IL-6, and considerably increase PDL-1 and PDL-2 expression at the protein level. In addition, no significant impact on HLA-DR and costimulatory molecules, both critical for immune response activation, was observed. Upregulation of PDL-1 and PDL-2 in f-LSCs could be involved in inhibition of lymphocyte proliferation and also associated with the decrease in IFN-γ and IL-17A levels as observed in female HT-derived T cells. The high expression of COX-2 enzyme, as a source of PGE2, could favor Th2-like cytokine secretion by inhibiting both Th1- and Th17-associated proliferation and, at the same time, by enhancing the production of Th2-associated cytokines such as IL-4. Upregulation of the chemotactic molecule MCP-1 could help the attraction of T cells into close proximity of f-LSCs, where high concentrations of cytotoxic factors synergistically may act to suppress T-cell function. Since f-LSC effects on immunocompetent cells were not MHC restricted and did not need to be matched with host HLAs, we cocultured human f-LSCs with allogenic PBMCs from healthy donors and female HT patients in the presence of anti-CD3 activating antibodies. We showed that, during coculture, f-LSCs failed to induce a full allogeneic T-lymphocyte response, suppressing up to 40% of PBMC proliferation as shown by cell cycle analysis and CFSE detection. When cell to cell contacts were blocked by transwell insertion, f-LSCs were less effective in inducing immunosuppression, suggesting the hypothesis that both surface and intracellular molecules were necessary to mount a proper immune response. We also demonstrated that, in the presence of f-LSCs, activated PBMCs were arrested in the G0/G1 cell cycle phase. This phenomenon, at the molecular level, was mediated by upregulation of the cyclin-dependent kinase inhibitor 1B (p27Kip1). No morphological changes, characteristic of apoptosis, were observed; nor was the possibility of triggering caspase-3 activity in PBMCs after coculture.

Having demonstrated that f-LSCs were able to inhibit allogeneic lymphocyte responses, we further explored the possible mechanisms underlying these effects. As an important effector cell, CD8^+^ T cells play a critical role in immune surveillance of the human body [38]. However, increasing evidence has demonstrated that CD8^+^ T cells also contribute to initiation and progression of several autoimmune diseases. Our data revealed that the CD4/CD8 ratio had been significantly inverted in healthy activated PBMCs after f-LSC interaction, proving a negative regulation action of f-LSCs on the CD8^+^ T subset. The conventional activation markers CD28 and PD-1 were reasonably downmodulated in the CD3^+^ subset while CD69 expression was significantly induced after coculture. It is important to note that CD69 was previously regarded as an activation marker of lymphocytes and a target of the canonical nuclear factor kappa-B (NF-kB) signaling [39, 40]. Recently, studies in CD69-deficient mice have revealed that it might act as a negative regulator and self-control molecule during lymphocyte activation and that in an immunoregulatory context late and sustained CD69 expression is promoted by noncanonical NF-kB signaling [41]. Therefore, stable CD69 expression defines cells with immunoregulatory properties. The activation status of PBMCs was also investigated by assessing expression of CD25. Flow cytometry analyses indicated a faint reduction in CD25 expression and preservation of the CD4^+^FoxP3^+^ pool in the presence or absence of f-LSCs. These data suggested that the suppressive effect of f-LSCs was not mediated through induction of regulatory T cells. Forty-eight hour cytokine prelicensing of f-LSCs with IL-6, IL-1β, and IFN-γ did not significantly improve their immunosuppressive performance on female HT PBMCs, suggesting that the proinflammatory background is not a necessary condition to induce the immunological effects of f-LSCs. The identification of helper T (Th) cell subsets has greatly improved our understanding of regulation of immune effector functions. Th subset differentiation is known to be regulated by the cytokine environment [42]. In our in vitro coculture model, f-LSCs seemed to influence the Th subset balance by altering the cytokine profile of T lymphocytes collected from female HT patients. By decreasing T-lymphocyte expression of IFN-γ, IL-17A, and RORγT, human f-LSCs might have attenuated the differentiation of naive CD4^+^ T cells into Th1 and Th17 effectors. In parallel, IL-4 expression was improved, shifting the total balance from Th1-driven responses to a more anti-inflammatory Th2 profile. ROR-γT is a key transcription factor for Th17 cell differentiation and important for IL-17A expression in vivo and in vitro. Our results showed downregulation of ROR-γ at the protein level, suggesting the capability of f-LSCs to also modulate the Th17 differentiation in favor of IL-4-producing Th2 cells. This phenomenon was more evident in T cells cocultured with f-LSCs and isolated from female patients. This effect may be due to an inflammatory background able to stimulate or maintain the immune process in AITD patients [43].

## Conclusions

In conclusion, we believe that due to all the features mentioned human f-LSCs are potent modulators of T cells. However, mechanisms exclusive to f-LSC-mediated immunomodulation warrant further investigation. In addition, as an easily accessible source of stem cells with a minimal and well-established surgical procedure, f-LSCs represent an excellent opportunity for use on a massive scale and as a cellular tool to induce tolerance in autoreactive lymphocytes from HT patients or other immunological disorders.

## Additional files

Additional file 1: Immunophenotyping of BM-MSCs. (A) The immunological profile of BM-MSCs was assessed by flow cytometry before (upper panel) and after 48 h of cytokine-treatment (IL-1β, IL-6, and IFNγ, 500 U/ml) (lower panel) using the same Abs for f-LSC immune characterization. All histogram plots include the percentage of expression of each protein as representative value of five independent experiments. (PDF 572 kb)

Additional file 2: f-LSCs arrest T-cell division. (A) One-way mixed leukocyte reaction (MLR) was carried out by seeding 2 × 10^5^ of responder healthy PBMCs into 48-well plates (Corning) and adding 1 × 10^5^ mytomycin-C pre-treated stimulator cells for a final volume of 500 μl of lymphocyte medium. Each responder and stimulator cell population was seeded in triplicate. When MLRs were cocultured with f-LSCs the allogenic activation was greatly reduced. Pictures are representative of five independent experiments. (B) Molecular detection of p27Kit and CCND1 mRNA by qRT-PCR. Results are shown as mean ± SD of three independent experiments; *p < 0.05. (PDF 138 kb)

Additional file 3: Inhibitory effect of BM-MSCs on PBMC proliferation. (A) PBMCs from healthy volunteers were stimulated with anti-CD3 for 72 h in the presence or absence of BM-MSCs (1:10 ratio). No activated PBMCs served as negative controls. Original magnification: 40×. Pictures are representative of at least five independent experiments (B). DNA content was assessed using PI staining and analyzed by flow cytometry. Analysis of cell cycle showed the 28 ± 2.7% of PBMCs after 72 h of activation in G2/S phase. The same cells in the presence of BM-MSCs became 15 ± 3.0% confirming the inhibitory action of BM-MSCs on PBMC proliferation. In the transwell system no significant inhibition of PBMC growing was observed (26.2 ± 3%). Unstimulated control cells were used as negative controls. FACS plots are representative of five experiments of identical design. Values on the bars are shown as mean ± SE; *p < 0.05. (PDF 488 kb)

Additional file 4: Jurkat cells as a positive control for apoptosis detection. (A) Untreated Jurkat cells (first row) or treated with activating anti-Fas for 4 or 24 h (second and third row, respectively) after AO/EB staining. The arrows indicate many apoptotic body formations, bright green dots in the nuclei as a consequence of chromatin condensation and nuclear fragmentation. Late apoptotic cells were observed with condensed and fragmented nuclei especially after 24 h of anti-Fas treatment. Original magnification: 40×. All pictures are representative of five independent experiments. (PDF 240 kb)

Additional file 5: Immunomodulation mechanism played from BM-MSCs on healthy activated PBMCs. (A) Acitive-caspase-3 detection by FACS revealed weak induction of the apoptotic cascade in activated healthy PBMCs after 72 h of coculture with BM-MSCs. FACS histogram plots are representative of five experiments of identical design. (B) The expression percentage of the three activation markers (CD28, PD-1, CD69) in activated healthy PBMCs were assessed by flow cytometry and reported in the histogram. Samples were run after 72 h of incubation with or without BM-MSCs. (C) The CD4/CD8 ratio was calculated from data obtained by flow staining in samples of healthy activated PBMCs alone and after coculture with BM-MSCs. (D) Detection of CD4^+^CD25^high^Foxp3^+^ fraction in purified T cells collected from healthy volunteers and stimulated with anti-CD3/CD28 for 3 days with or without BM-MSCs. In all experiments unstimulated PBMCs were used as negative controls. Data are presented as means ± SE in each histogram; *p < 0.05, **p < 0.02. (PDF 381 kb)

## Abbreviations

AIRE: Autoimmune regulator
AITD: Autoimmune thyroid disease
A-MSC: Adipose-derived mesenchymal stem cell
AO/EB: Acridine orange/ethidium bromide
bFGF: Basic fibroblast growth factor
BM-MSC: Bone marrow-derived mesenchymal stem cell
BSA: Bovine serum albumin
CB-MSC: Cord blood-derived mesenchymal stem cell
CFSE: Carboxyfluorescein succinimidyl ester
COX-2: Cyclooxygenase-2
DMEM: Dulbecco’s modified Eagle’s medium
EC-FBS: Embryonic stem cell-tested fetal bovine serum
f-LSC: Fibroblast-like limbal stem cell
HGF: Hepatocyte growth factor
HLA: Human leukocyte antigen
HT: Hashimoto’s thyroiditis
IDO: Indoleamine-pyrrole 2,3-dioxygenase
IFN: Interferon
IL: Interleukin
ITS: Insulin-transferrin-selenium
LESC: Limbal epithelial stem cell
LSCD: Limbal stem cell deficiency
MCP-1: Monocyte chemotactic protein 1
MHC: Major histocompatibility
MSC: Mesenchymal stem cell
PBMC: Peripheral blood mononuclear cell
PBS: Phosphate-buffered saline
PDL-1/2: Programmed death-ligand 1/2
PGE2: Prostaglandin E2
PI: Propidium iodide
P-MSC: Placenta- derived mesenchymal stem cell
qRT-PCR: Quantitative reverse-transcription polymerase chain reaction
Tg: Thyroglobulin
TGF: Transforming growth factor
TPO: Thyroperoxidase
TSH-R: Thyroid stimulating hormone receptor
