# Supplementary figures and images for: Human limbal fibroblast-like stem cells induce immune-tolerance in autoreactive T lymphocytes from female patients with Hashimoto’s thyroiditis
Source: Stem Cell Res Ther. 2017 Jul 3;8:154. doi: 10.1186/s13287-017-0611-5 (PMC5496215; doi:10.1186/s13287-017-0611-5)

**A****HLA-DR****PD****PDL-1****PDL-2****untreated**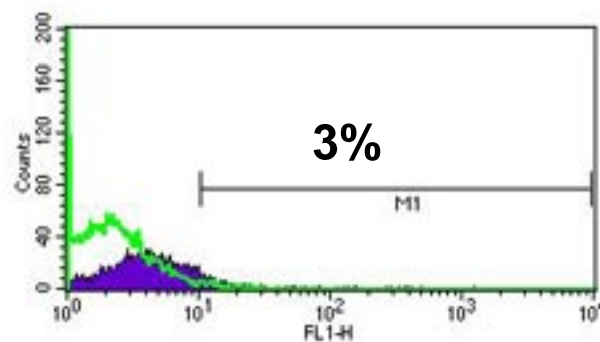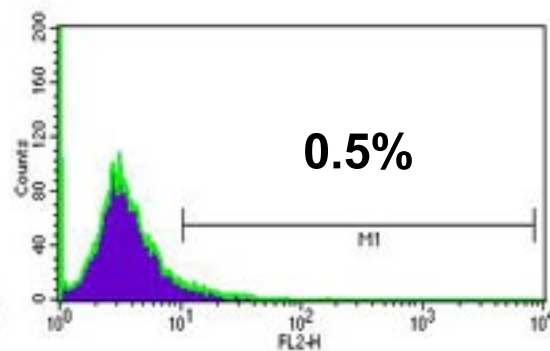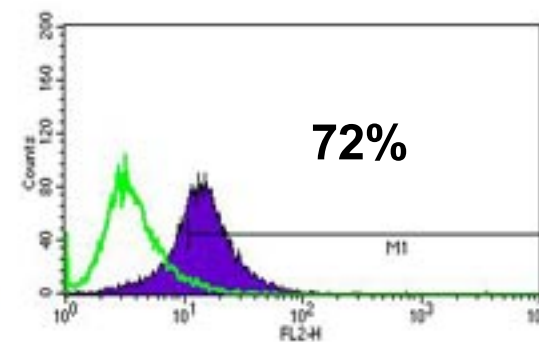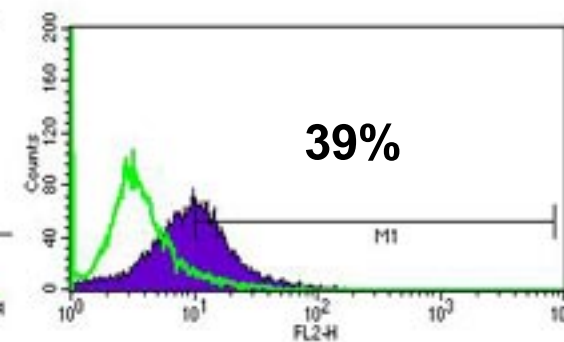**B7H4****CD80****CD86**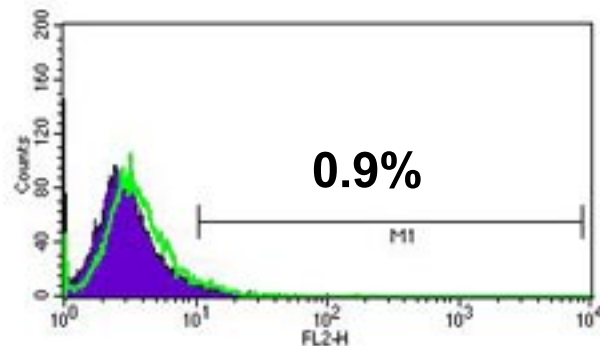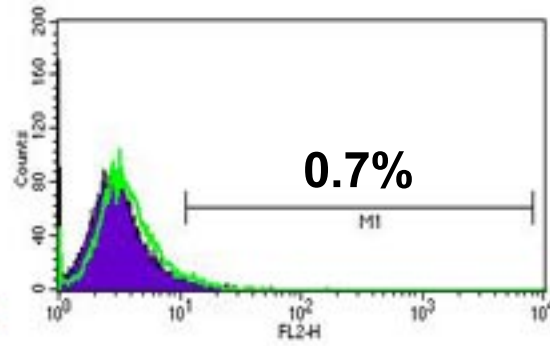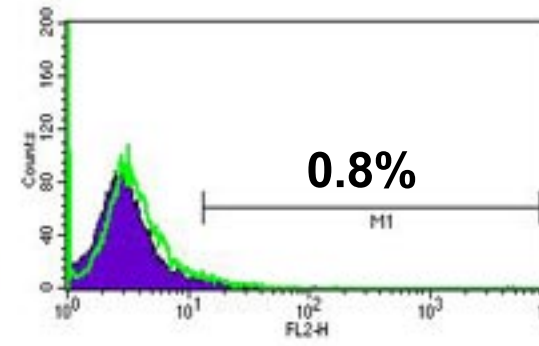**HLA-DR****PD****PDL-1****PDL-2****+ cytokines**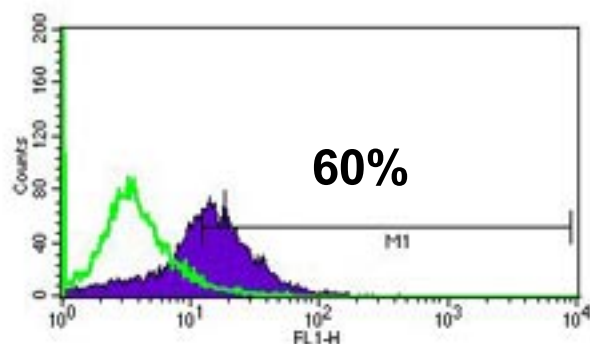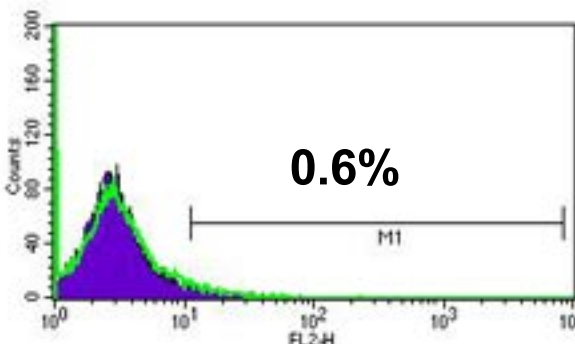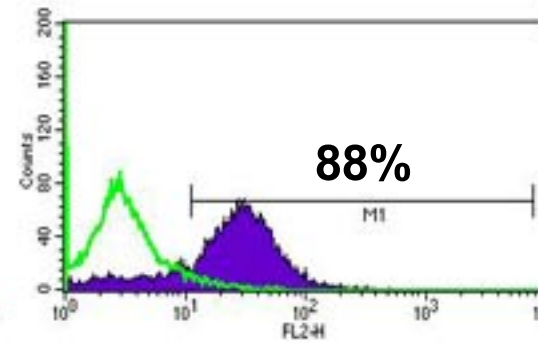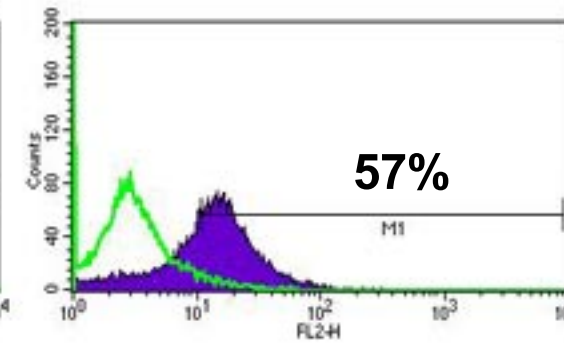**B7H4****CD80****CD86**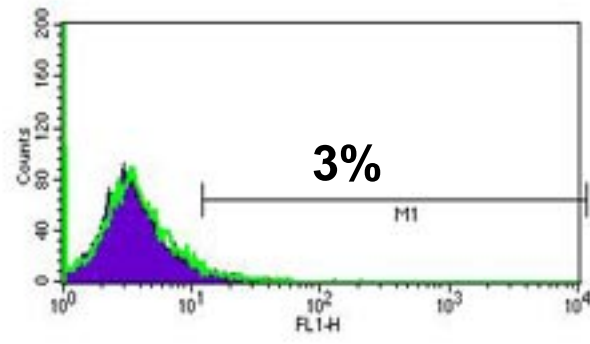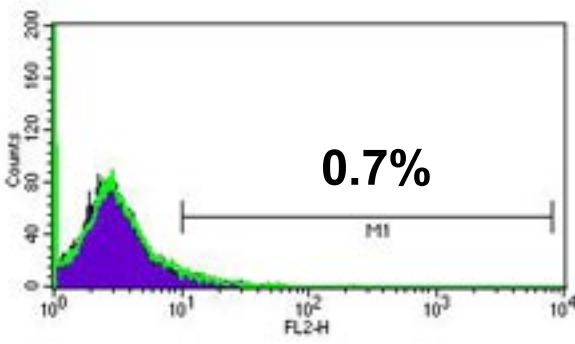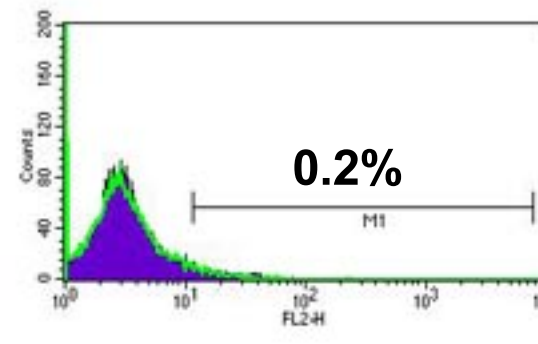

Supplement: Supplementary file 1 — Immunophenotyping of BM-MSCs. (A) The immunological profile of BM-MSCs was assessed by flow cytometry before (upper panel) and after 48 h of cytokine-treatment (IL-1β, IL-6, and IFNγ, 500 U/ml) (lower panel) using the same Abs for f-LSC immune characterization. All histogram plots include the percentage of expression of each protein as representative value of five independent experiments. (PDF 572 kb) [file 13287_2017_611_MOESM1_ESM.pdf]

**A**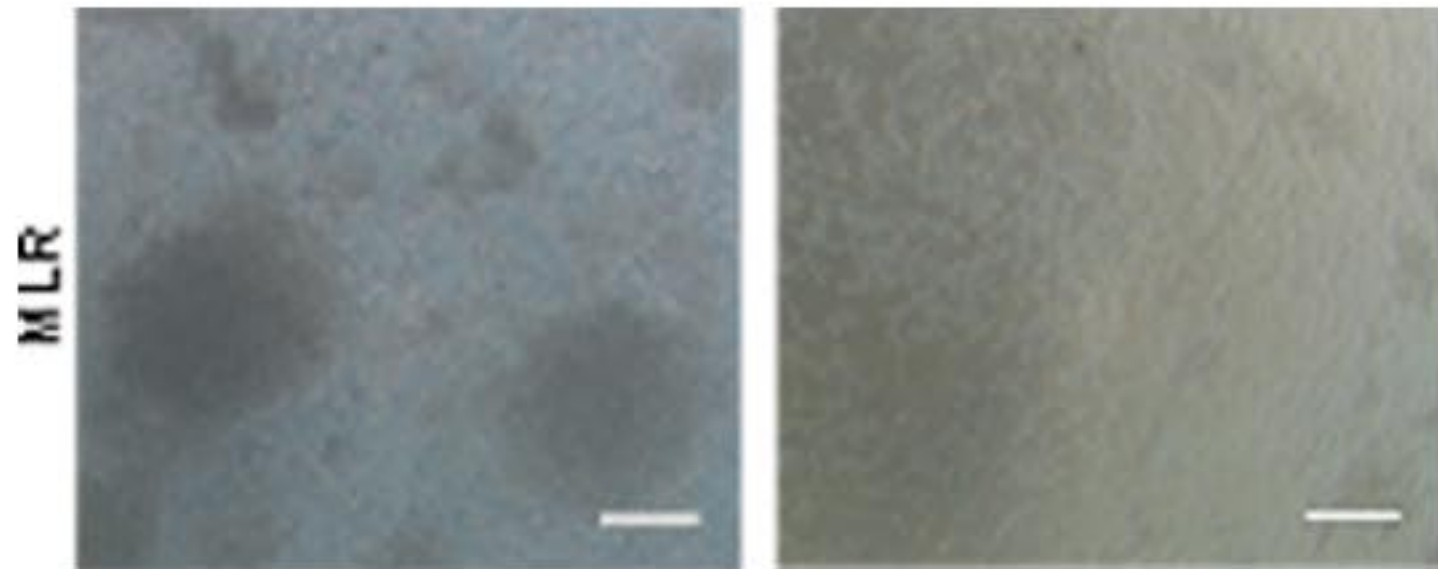**B**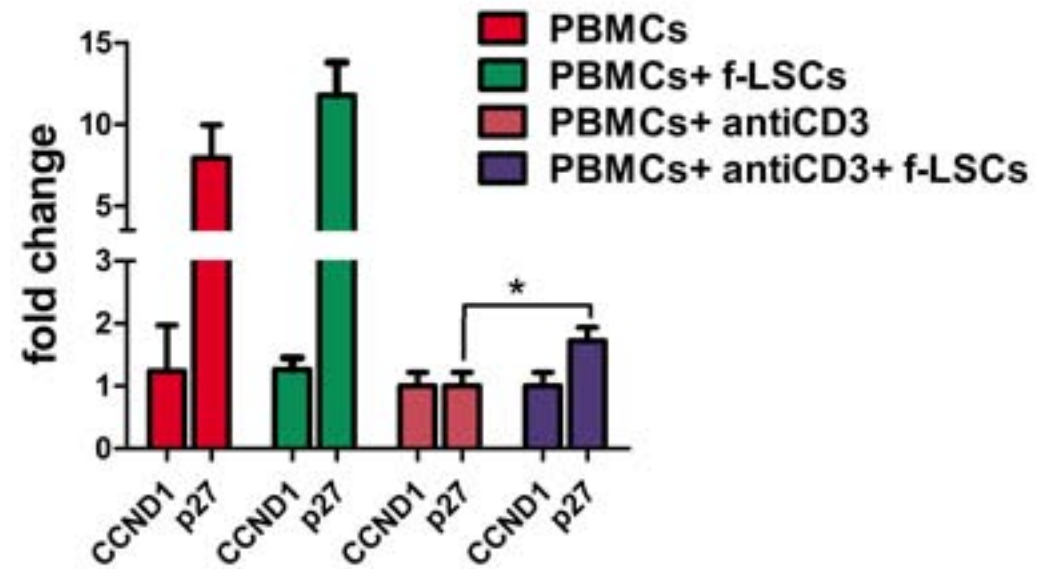

Supplement: Supplementary file 2 — f-LSCs arrest T-cell division. (A) One-way mixed leukocyte reaction (MLR) was carried out by seeding 2 × 10^5^ of responder healthy PBMCs into 48-well plates (Corning) and adding 1 × 10^5^ mytomycin-C pre-treated stimulator cells for a final volume of 500 μl of lymphocyte medium. Each responder and stimulator cell population was seeded in triplicate. When MLRs were cocultured with f-LSCs the allogenic activation was greatly reduced. Pictures are representative of five independent experiments. (B) Molecular detection of p27Kit and CCND1 mRNA by qRT-PCR. Results are shown as mean ± SD of three independent experiments; *p < 0.05. (PDF 138 kb) [file 13287_2017_611_MOESM2_ESM.pdf]

**A**

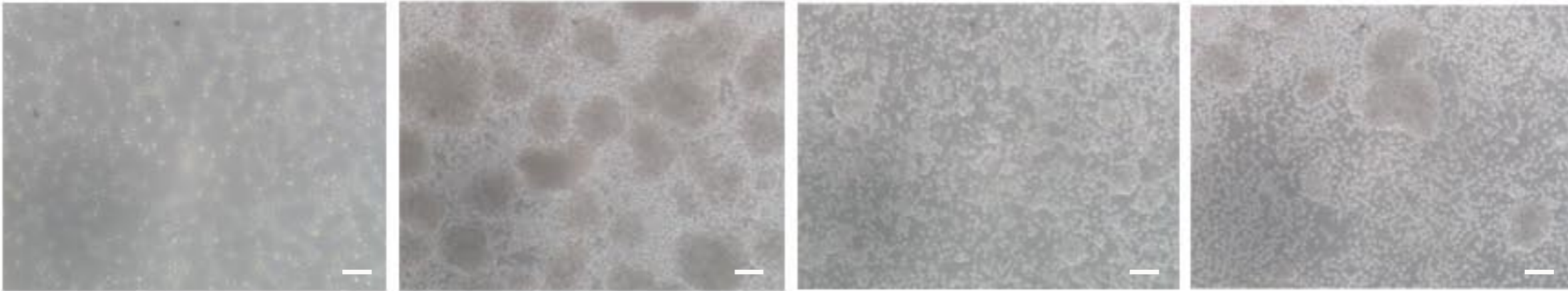

|           |   |   |   |   |
|-----------|---|---|---|---|
| PBMCs     | + | + | + | + |
| anti-CD3  | - | + | + | + |
| BM-MSCs   | - | - | + | + |
| transwell | - | - | - | + |

**B**

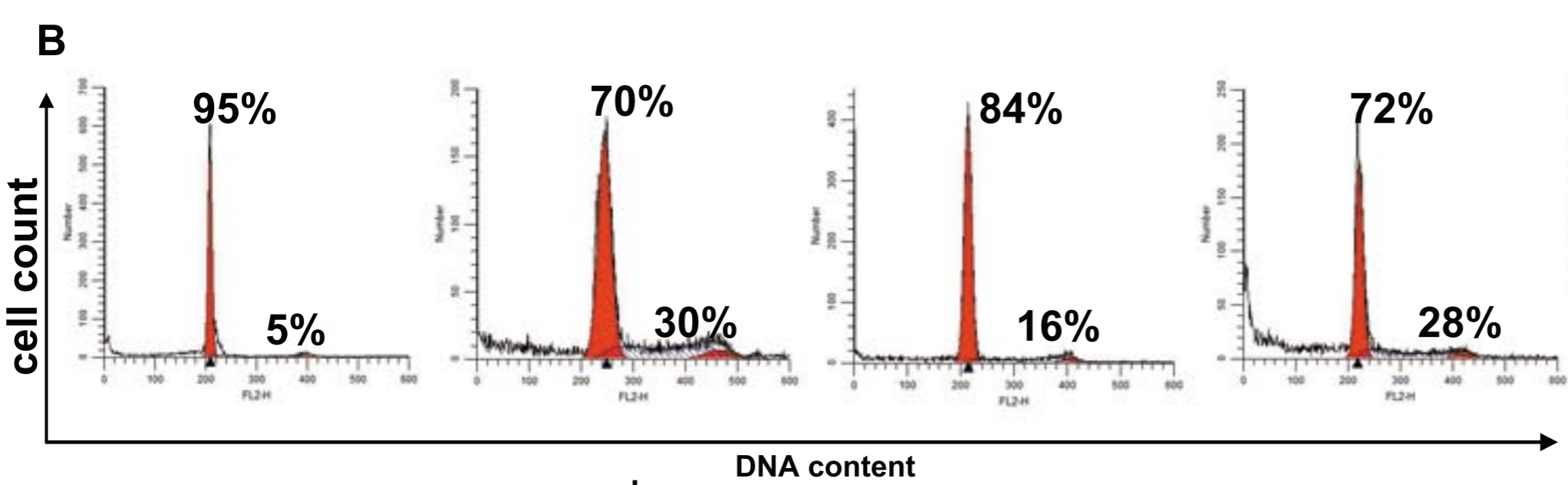

|           |   |   |   |   |
|-----------|---|---|---|---|
| PBMCs     | + | + | + | + |
| anti-CD3  | - | + | + | + |
| BM-MSCs   | - | - | + | + |
| transwell | - | - | - | + |

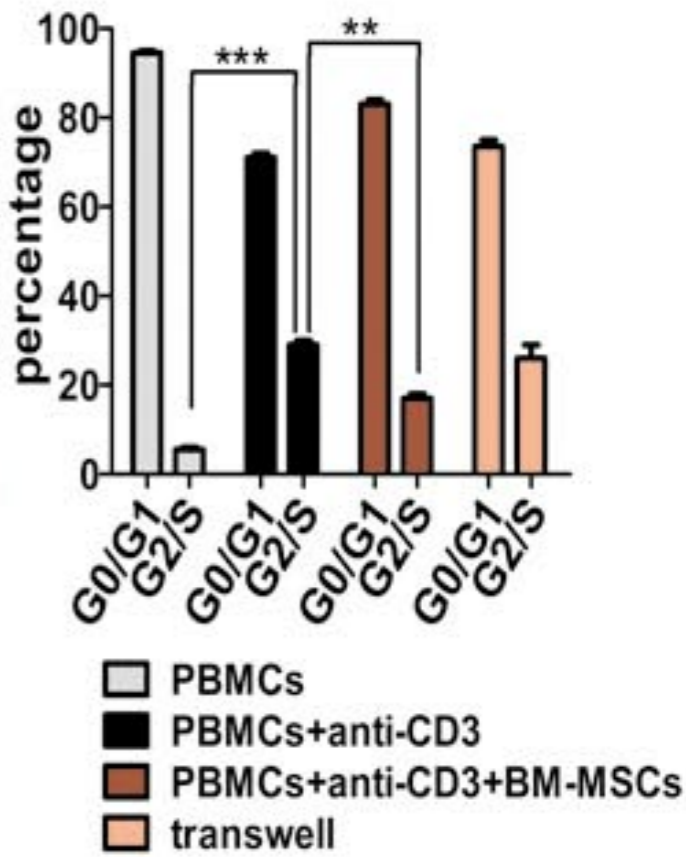

Supplement: Supplementary file 3 — Inhibitory effect of BM-MSCs on PBMC proliferation. (A) PBMCs from healthy volunteers were stimulated with anti-CD3 for 72 h in the presence or absence of BM-MSCs (1:10 ratio). No activated PBMCs served as negative controls. Original magnification: 40×. Pictures are representative of at least five independent experiments (B). DNA content was assessed using PI staining and analyzed by flow cytometry. Analysis of cell cycle showed the 28 ± 2.7% of PBMCs after 72 h of activation in G2/S phase. The same cells in the presence of BM-MSCs became 15 ± 3.0% confirming the inhibitory action of BM-MSCs on PBMC proliferation. In the transwell system no significant inhibition of PBMC growing was observed (26.2 ± 3%). Unstimulated control cells were used as negative controls. FACS plots are representative of five experiments of identical design. Values on the bars are shown as mean ± SE; *p < 0.05. (PDF 488 kb) [file 13287_2017_611_MOESM3_ESM.pdf]

**C**

**untreated**

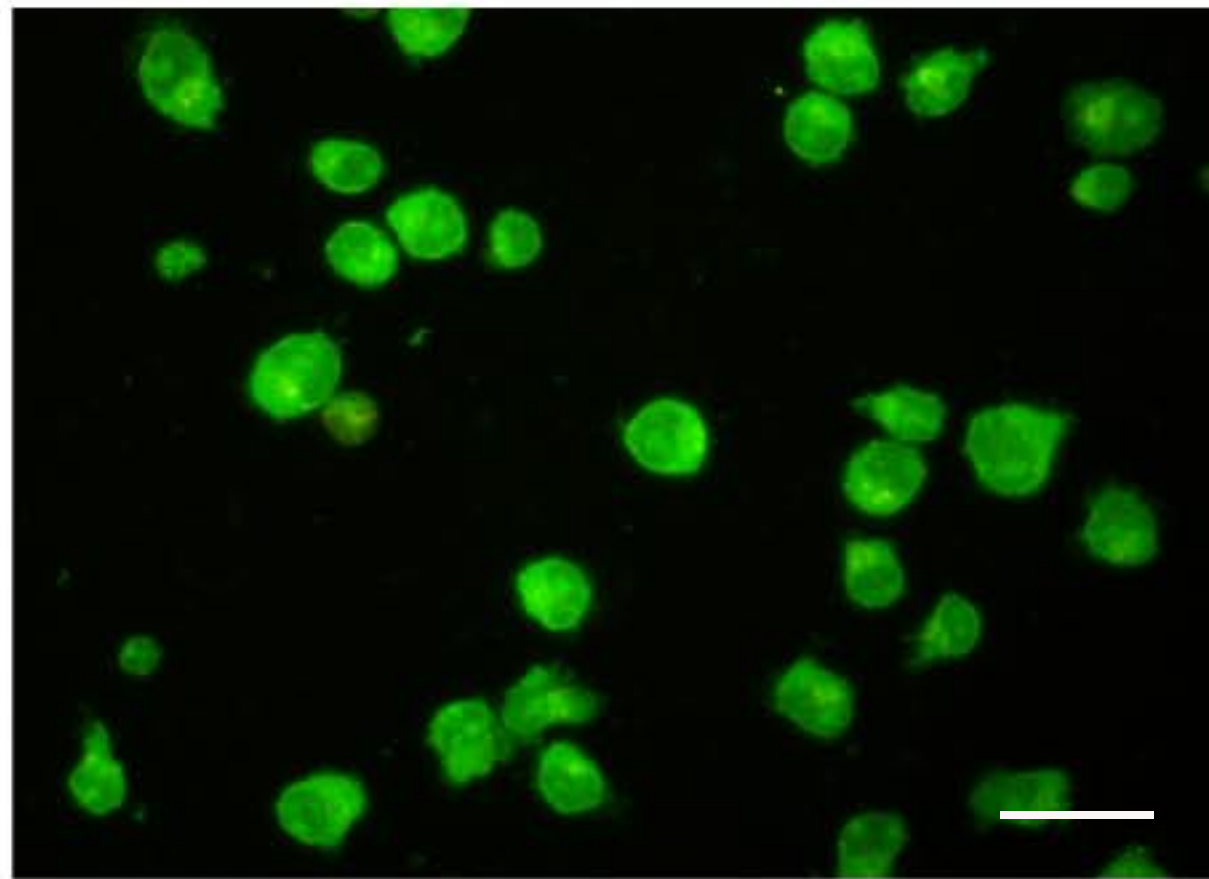

**+CH11 4h**

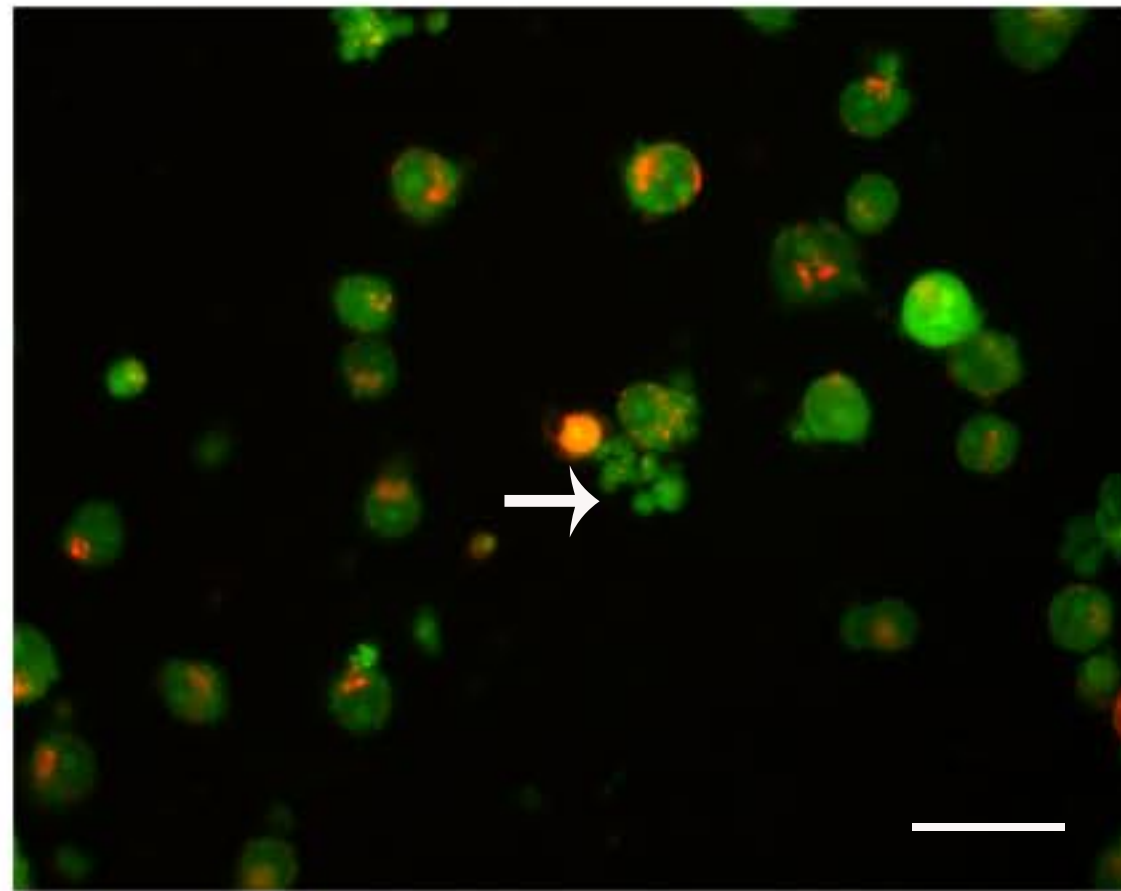

**+CH11 24h**

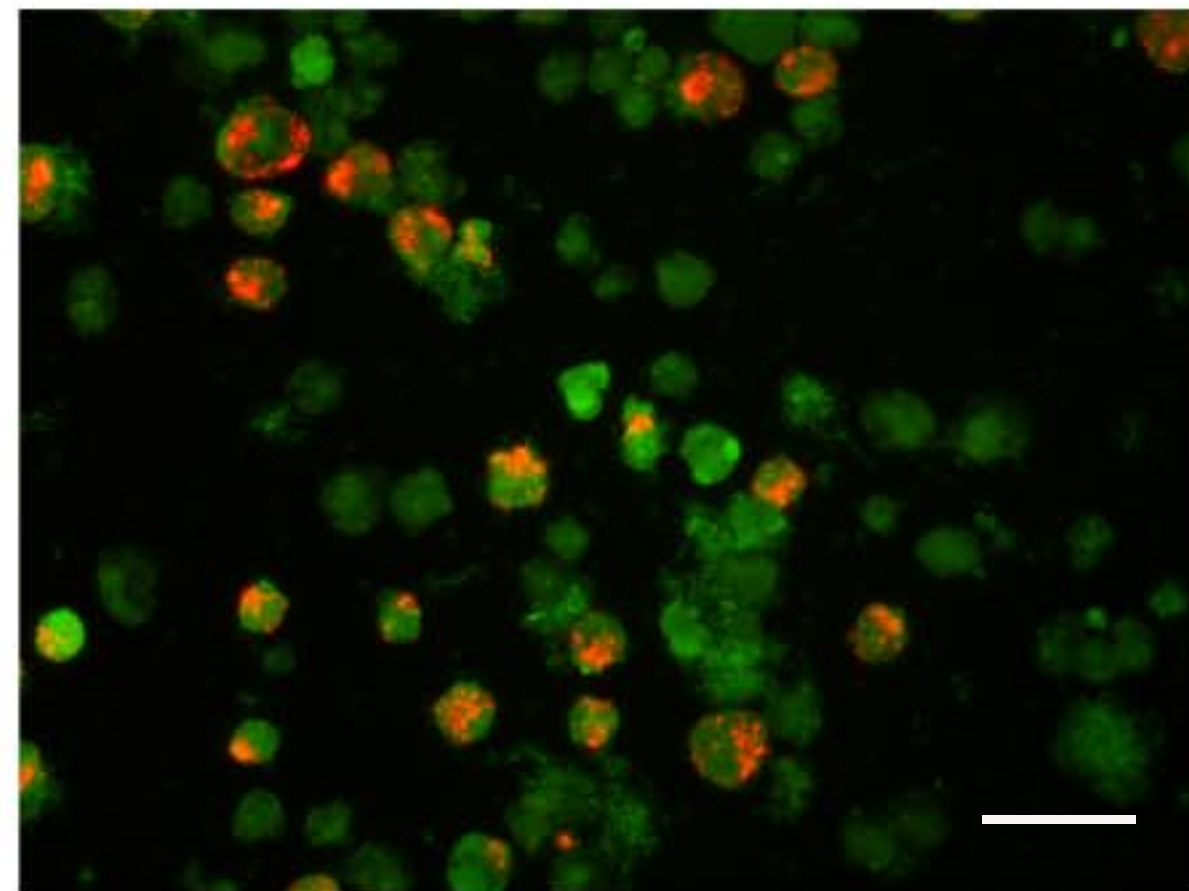

Supplement: Supplementary file 4 — Jurkat cells as a positive control for apoptosis detection. (A) Untreated Jurkat cells (first row) or treated with activating anti-Fas for 4 or 24 h (second and third row, respectively) after AO/EB staining. The arrows indicate many apoptotic body formations, bright green dots in the nuclei as a consequence of chromatin condensation and nuclear fragmentation. Late apoptotic cells were observed with condensed and fragmented nuclei especially after 24 h of anti-Fas treatment. Original magnification: 40×. All pictures are representative of five independent experiments. (PDF 240 kb) [file 13287_2017_611_MOESM4_ESM.pdf]

**A**

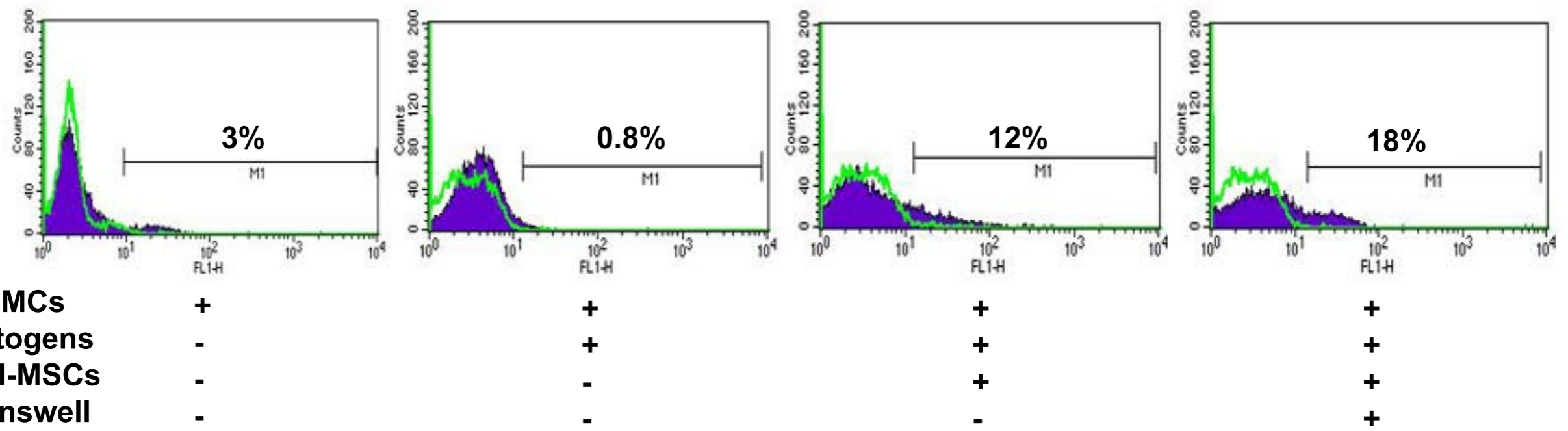

**B**

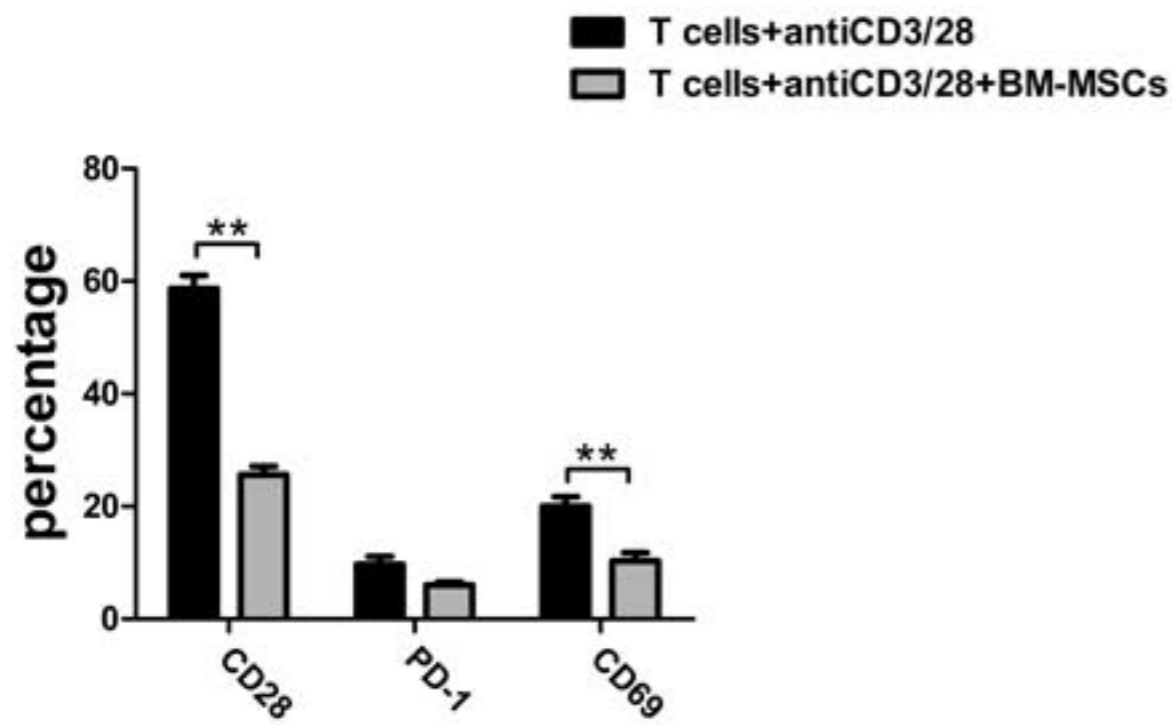

**C**

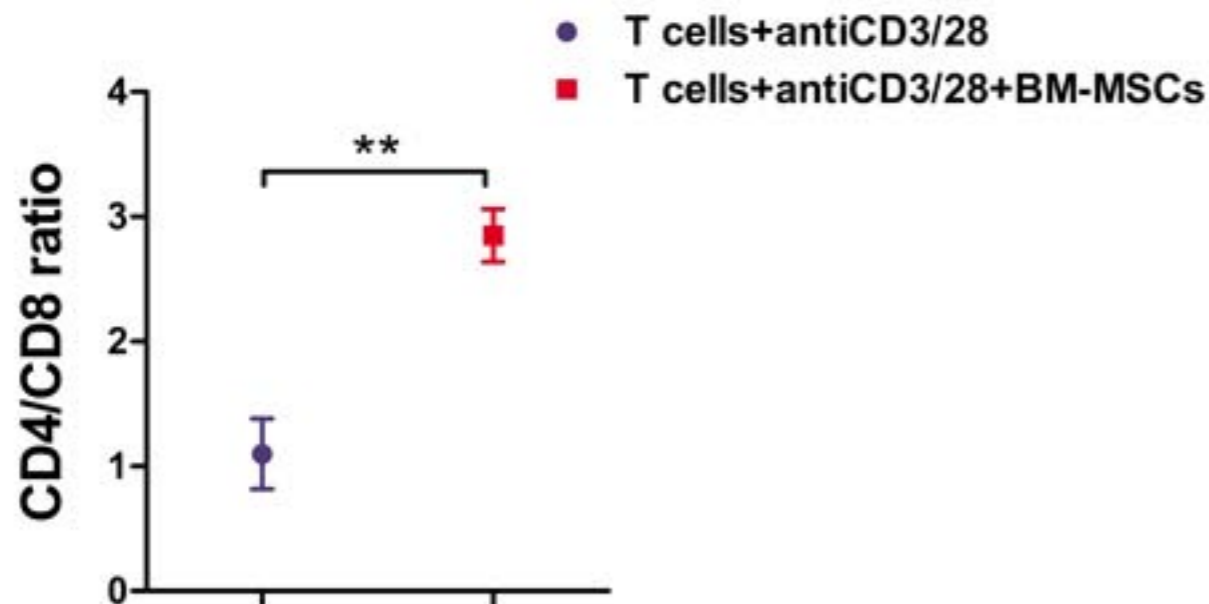

**D**

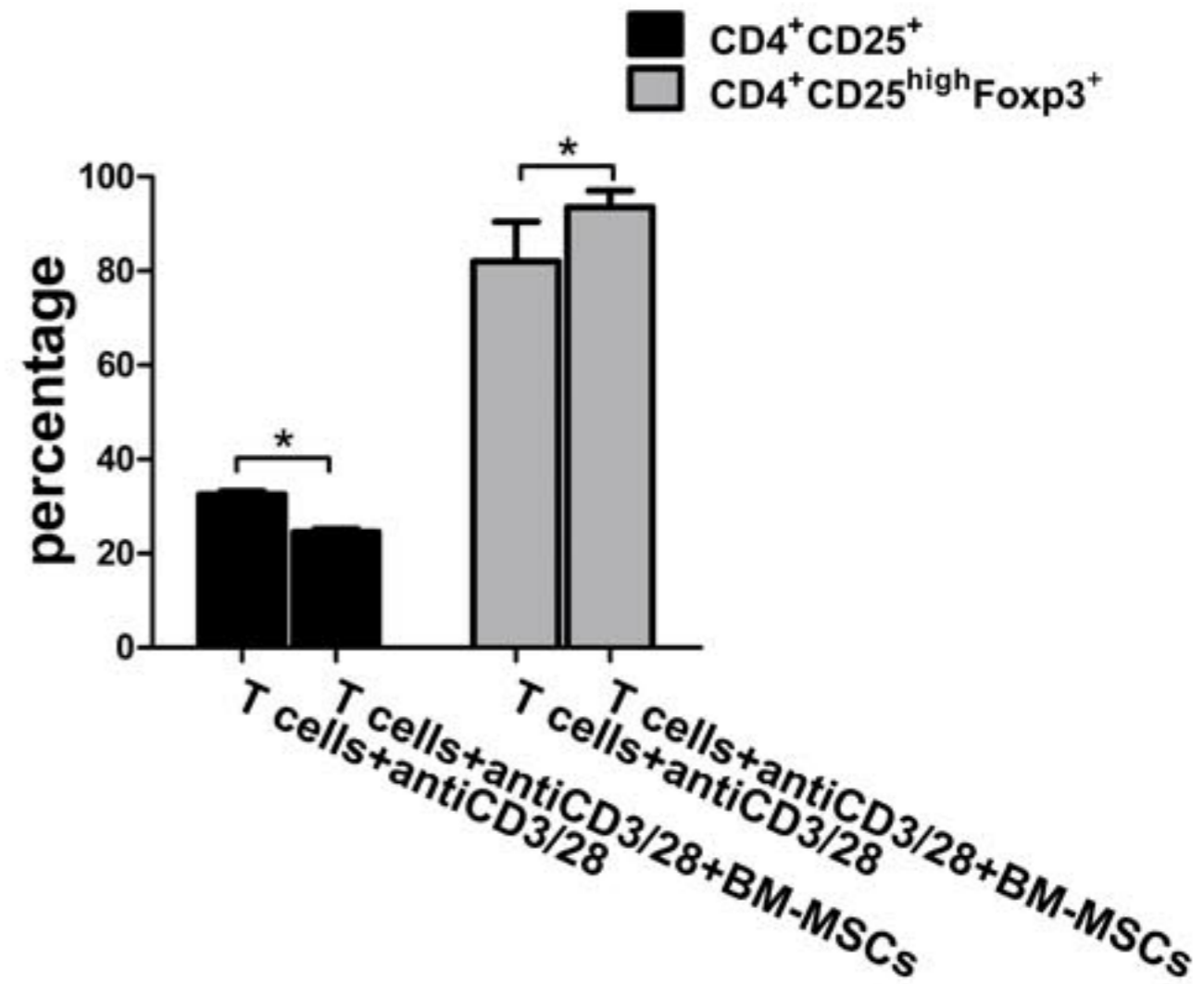

Supplement: Supplementary file 5 — Immunomodulation mechanism played from BM-MSCs on healthy activated PBMCs. (A) Acitive-caspase-3 detection by FACS revealed weak induction of the apoptotic cascade in activated healthy PBMCs after 72 h of coculture with BM-MSCs. FACS histogram plots are representative of five experiments of identical design. (B) The expression percentage of the three activation markers (CD28, PD-1, CD69) in activated healthy PBMCs were assessed by flow cytometry and reported in the histogram. Samples were run after 72 h of incubation with or without BM-MSCs. (C) The CD4/CD8 ratio was calculated from data obtained by flow staining in samples of healthy activated PBMCs alone and after coculture with BM-MSCs. (D) Detection of CD4+CD25highFoxp3+ fraction in purified T cells collected from healthy volunteers and stimulated with anti-CD3/CD28 for 3 days with or without BM-MSCs. In all experiments unstimulated PBMCs were used as negative controls. Data are presented as means ± SE in each histogram; *p < 0.05, **p < 0.02. (PDF 381 kb) [file 13287_2017_611_MOESM5_ESM.pdf]
